# Gene genealogies for genetic association mapping, with application to Crohn's disease

**DOI:** 10.3389/fgene.2013.00260

**Published:** 2013-12-02

**Authors:** Kelly M. Burkett, Celia M. T. Greenwood, Brad McNeney, Jinko Graham

**Affiliations:** ^1^Department of Statistics and Actuarial Science, Simon Fraser UniversityBurnaby, BC, Canada; ^2^Department of Epidemiology, Biostatistics and Occupational Health, McGill UniversityMontreal, QC, Canada; ^3^Department of Oncology, Department of Epidemiology, Biostatistics and Occupational Health, and Division of Cancer Epidemiology, McGill UniversityMontreal, QC, Canada; ^4^Lady Davis Institute for Medical Research, Jewish General HospitalMontreal, QC, Canada

**Keywords:** coalescent model, gene genealogy, Markov chain Monte Carlo, fuzzy *p*-value, association study, Crohn's disease

## Abstract

A gene genealogy describes relationships among haplotypes sampled from a population. Knowledge of the gene genealogy for a set of haplotypes is useful for estimation of population genetic parameters and it also has potential application in finding disease-predisposing genetic variants. As the true gene genealogy is unknown, Markov chain Monte Carlo (MCMC) approaches have been used to sample genealogies conditional on data at multiple genetic markers. We previously implemented an MCMC algorithm to sample from an approximation to the distribution of the gene genealogy conditional on haplotype data. Our approach samples ancestral trees, recombination and mutation rates at a genomic focal point. In this work, we describe how our sampler can be used to find disease-predisposing genetic variants in samples of cases and controls. We use a tree-based association statistic that quantifies the degree to which case haplotypes are more closely related to each other around the focal point than control haplotypes, without relying on a disease model. As the ancestral tree is a latent variable, so is the tree-based association statistic. We show how the sampler can be used to estimate the posterior distribution of the latent test statistic and corresponding latent *p*-values, which together comprise a fuzzy *p*-value. We illustrate the approach on a publicly-available dataset from a study of Crohn's disease that consists of genotypes at multiple SNP markers in a small genomic region. We estimate the posterior distribution of the tree-based association statistic and the recombination rate at multiple focal points in the region. Reassuringly, the posterior mean recombination rates estimated at the different focal points are consistent with previously published estimates. The tree-based association approach finds multiple sub-regions where the case haplotypes are more genetically related than the control haplotypes, and that there may be one or multiple disease-predisposing loci.

## 1. Introduction

The gene genealogy describes the relationships among haplotypes sampled from a population. For a genomic region undergoing recombination, different locations within that region have different ancestral origins and therefore different ancestries. The gene genealogy for these recombining regions may be represented as a graph, called the ancestral recombination graph (ARG). However, at each site in the region, the ancestral history of that genomic position can be represented as a tree; this marginal tree can be extracted from the full ARG.

The concept of the gene genealogy has been useful in the estimation of population genetic parameters. It is worth noting, however, that the concept also has potential application in finding disease-predisposing genetic variants. Since haplotypes from case individuals are genetically more closely related to each other at the site of a disease-predisposing mutation, their haplotypes would appear to cluster together in the marginal ancestral tree at the site of the mutation. The ancestry also offers a useful data reduction strategy. Cluster membership defined by the ancestral tree summarizes the genotypic similarity across multiple markers and association of disease with cluster membership can be tested, rather than association with each of the marker loci individually.

There has been much interest in incorporating the ancestral history of a sample of sequences into association study methodology. However, the time scale for the gene genealogy is on the order of tens of thousands of years, and there is therefore no way to know the true underlying gene genealogy for a random sample of sequences. Ancestry-based association methods must handle this uncertainty appropriately. The genetic marker data reflects the underlying but unknown genealogy and therefore it can be used to estimate the distribution of the gene genealogy. Many approaches have used phylogenetic methods to first impute a single marginal tree for a region based on the observed marker data and then used the imputed tree to define clusters or clades (for example, Templeton et al., 1987; Durrant et al., 2004; Bardel et al., 2005; Mailund et al., 2006; Kimmel et al., 2008). Other approaches have used statistical clustering techniques to cluster the haplotypes (Waldron et al., 2006; Igo et al., 2009; Jin et al., 2010) or to sample multiple likely clusterings (Molitor et al., 2003). With any of these approaches, cluster membership is then tested for association with the phenotype. Minichiello and Durbin (2006) and Adhikari et al. (2012) proposed heuristic rule-based algorithms to sample from an approximation to the ARG across a region. Each sampled graph is then tested for association and the resulting statistic is averaged over the sample. There are two reasons why these approaches are not optimal. First, many of these approaches use a single imputed tree and treat the tree as if it were known; therefore, tree uncertainty is not addressed. Second, even for those approaches that sample multiple trees or approximations to the ARGs, the models used to sample the trees and graphs are not informed by population genetic models like the coalescent (Kingman, 1982; Hudson, 1990), which gives a prior distribution for the shape and branch lengths of gene genealogies.

In order to handle tree uncertainty, we previously implemented an algorithm, called sampletrees, that treats the marginal tree at a genomic position as a latent variable and uses Markov chain Monte Carlo (MCMC) to sample realizations of the tree, recombination and mutation rates conditional on haplotype data at multiple markers (Burkett et al., 2013a). Provided that the underlying model for the ancestry is applicable, any tree-based association statistic can then be computed on the sampled trees in order to estimate the posterior distribution of the association statistic conditional on the data.

In this work, we present a proof-of-concept demonstration of the usefulness of genealogic trees in fine-mapping of complex traits. We apply a tree-based association method that relies on ancestral trees sampled with sampletrees. We first briefly review the sampletrees model and the MCMC algorithm. We then introduce a tree-based association statistic that measures the degree to which case haplotypes are more closely related than control haplotypes, without relying on a disease penetrance model. Since the genealogical tree is a latent variable, so is the tree-based association statistic. We subsequently show how the strength of the association signal and the uncertainty associated with the latent variable can be expressed by the fuzzy *p*-value (Thompson and Geyer, 2007), which is the distribution of latent *p*-values corresponding to the latent tree-based association statistic evaluated at each of the sampled trees.

We illustrate this analytic approach using the publicly-available “crohn” dataset, which was analyzed by Rioux et al. (2001), and is available in the R gap package (Zhao, 2013). The data consist of genotypes at multiple SNP markers in a 500 kb region on chromosome 5 for a sample of trios comprising a child affected with Crohn's disease and his or her two parents. Rioux et al. (2001) found significant associations at 11 loci spanning 200 kb and including multiple genes; the risk alleles at the 11 loci have been collectively labeled the IBD5 risk haplotype. Since the original publication, association of Crohn's disease with the IBD5 risk haplotype has been replicated in multiple studies (see Cooney and Jewell; 2009 and Barrett and Chandra, 2011 for reviews). However, because of the strong linkage disequilibrium (LD) in the region, the SNPs in IBD5 give essentially equivalent association information (Waller et al., 2006) and the location of the true disease-predisposing variant(s) remains unknown. Using our methods, we estimate the posterior distribution of the tree-based association statistic and recombination rates at 100 different focal points in the 500 kb region. For each focal point, we compute the fuzzy *p*-value of the association statistic and use the median of the latent *p*-value distribution as a measure of the strength of association.

## 2. Materials and methods

### 2.1. The SAMPLETREES algorithm

We previously implemented an MCMC algorithm to sample ancestral trees and population genetic parameters conditional on multi-marker data. It is based on the sampler that was outlined in Zöllner and Pritchard (2005), with some changes that are described in detail in Burkett et al. (2013a). Our sampler, implemented in the C++ program sampletrees, is available at http://stat.sfu.ca/statgen/research/sampletrees.html. An R package for sampletrees is currently under development. In this section, we give a brief description of the sampler.

Letting a “focal point" be a genomic position of interest, recall that at each site or focal point in a genomic region, the ancestral history of the site is described by a marginal ancestral tree that can be extracted from the ARG of that region. The approach used in Zöllner and Pritchard (2005), as well as in our implementation, is to sample ancestral trees at a focal point, rather than sample ARGs that capture the full ancestral history of the region. Hence, to construct ancestral histories across a larger region, trees are sampled from their marginal (as opposed to joint) posterior distributions.

The MCMC algorithm samples T_*x*_, the tree structure and internal node times, at focal point *x* conditional on genetic marker data *G* from the posterior distribution *f*(T_*x*_|G). In order to model *f*(T_*x*_|G), the distribution of the tree conditional on the marker data, additional latent variables corresponding to the haplotypes at the internal nodes of the tree, recombination break points, and mutation and recombination rates are added to the model. The recombination event rate, ρ/2, is the rate of recombination per unit of coalescence time, per pair of adjacent base pairs. The mutation event rate, θ/2, is the rate of mutation of an ascertained SNP, per unit of coalescence time. The posterior distribution can then be written in terms of standard population genetic models of sequence mutation, recombination and the coalescent process.

Letting A represent the augmented data including the additional latent variables, and *Q_i_*(Ã|A) be the *i*th proposal distribution, at the *j*th iteration of the MCMC algorithm, Ã is accepted as the *j*th sample with probability determined by the Metropolis-Hastings ratio

$$\alpha =\frac{f(\tilde{\text{A}}|\text{G}){\text{Q}}_{\text{i}}({\text{A}}^{\left(\text{j }-\text{1}\right)}|\tilde{\text{A}})}{f({\text{A}}^{\left(\text{j }-\text{1}\right)}|\text{G}){\text{Q}}_{\text{i}}(\tilde{\text{A}}|{\text{A}}^{\left(\text{j }-\text{1}\right)})}.$$

Each proposal distribution proposes new values for a subset of the augmented data A. The five proposal distributions modify: (1) the mutation rate, (2) the recombination rate, (3) the data at an internal node of the tree, and (4) and (5) modify the topology of the tree. At each step of the MCMC algorithm, one of the five proposal types is applied. The proposal distribution to apply at a given step is randomly sampled according to a set of user-supplied probabilities.

For the specified number of MCMC samples (*N*), sampletrees returns the tree (topology and node times), the mutation and recombination rates. Due to the large tree and haplotype file sizes, we recommend thinning the Markov chain by returning trees at periodic intervals. In addition, sampling ancestral trees is computationally intensive and, as with all MCMC algorithms, convergence issues and slow mixing are a possibility; it is therefore important to use MCMC convergence diagnostic techniques to evaluate results. Additional details about our sampler can be found in Burkett et al. (2013a,b).

### 2.2. Tree-based association statistic

**Figure 1:**
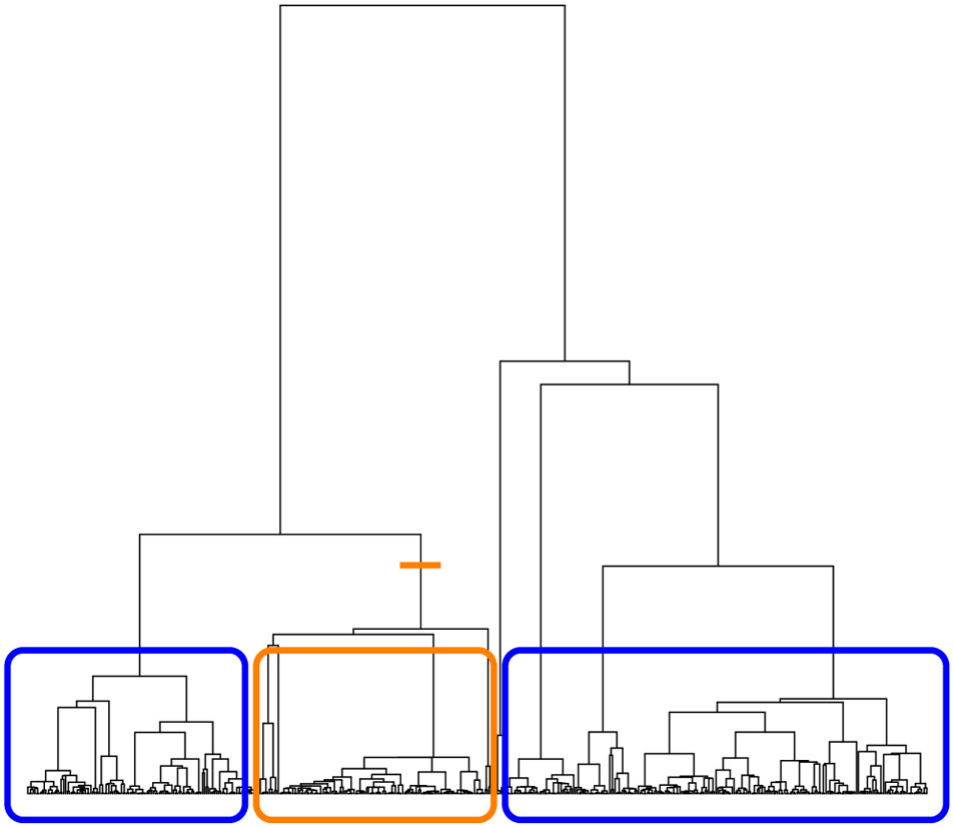
**Illustration of bipartition clustering.** The tree is cut at an internal branch of the tree (orange line). The tips of the tree descending from that branch form one group (orange box) and the tips of the tree that do not descend from that branch form the second group (two blue boxes).

On each of the sampled trees returned by the sampletrees function, we can compute a tree-based association statistic summarizing the degree to which haplotypes from individuals with similar trait values are related. We are particularly interested in statistics that are non-parametric; that is, statistics that do not require specifying a disease model. With respect to the ancestral tree of the disease mutation, haplotypes from case individuals would show evidence of being more closely related if they tend to preferentially coalesce or cluster with each other rather than with haplotypes from controls. We therefore use the tree to define clusterings of the tips. Since many different clusterings can be induced by a single tree, we focus on bipartition clusterings, as illustrated in Figure 1. Each internal branch of the tree induces a partition of the data into two groups: tips that descend from a given branch form one group and tips that do not descend from the branch form the second group.

If a hypothetical disease-predisposing mutation occurred on one branch, descendants of that branch will all carry the mutation and will also be more likely to be from case individuals. The cases should appear to cluster together in the group defined by the branch where the disease mutation occurred. Therefore, to determine if haplotypes from cases tend to cluster in an ancestral tree, we measure association between cluster membership and disease status for all eligible bipartitions of the tree using the absolute value of the Pearson correlation coefficient. We chose to define a bipartition as eligible if it leads to a cluster containing at least 5% of the total number of haplotypes sampled. This choice of minimum cluster size is arbitrary, but it avoids the need to compute the association on clusters that are too small to be interesting. The ancestry association statistic for the *i*th tree, *T_i_*, is the maximum, across all eligible clusterings, of the absolute correlation between disease status and cluster membership. This statistic is similar to the association statistic described in Minichiello and Durbin (2006); however, they do not use a lower bound on the number of tips in a cluster.

### 2.3. Fuzzy *p*-value

The fuzzy *p*-value (Thompson and Geyer, 2007) can be used as a measure of the strength of association when the test statistic of interest, *T*, is a function of a latent variable. For a tree-based test statistic capturing an increased clustering of the case haplotypes, the posterior distribution of that statistic will differ from the prior distribution. Here, the posterior distribution of a latent variable refers to the distribution conditional on the marker data, whereas the prior distribution refers to the distribution unconditional on the marker data. To measure the discrepancy between the posterior and prior distributions, we use the posterior distribution of latent *p*-values, which is called the fuzzy *p*-value. The fuzzy *p*-value expresses both the strength of evidence and the uncertainty associated with the latent variables.

For a realization of the tree statistic, *T^c^_j_*, sampled from the posterior distribution, the latent *p*-value measures how compatible this statistic is with the prior distribution. We take *T^c^_j_* to be the maximum across bipartitions of the correlation between cluster membership and case status. In the context of latent gene genealogies of genomic focal points, we can assume the neutral coalescent model (Kingman, 1982; Hudson, 1990) as the prior distribution for the ancestral tree unconditional on the data. To estimate the prior distribution of the test statistic, *M* trees are sampled from the neutral coalescent model and the tree-based association statistic, the maximum correlation statistic, is computed on each tree, leading to the unconditional sample T^*u*^ = (*T*^*u*^_1_,*T*^*u*^_2_, … *T*^*u*^_*M*_). For *T^c^_j_*, the *j*th maximum correlation statistic sampled from the posterior distribution using sampletrees, the latent *p*-value is

$$p_{j}=\frac{\sum_{\text{i=1}}^{\text{i=M}}1[T_{i}^{u}\geq T_{j}^{c}]}{M}.$$

The latent *p*-value is computed for all trees sampled from the posterior distribution, leading to a distribution of latent *p*-values, (*p*_1_,*p*_2_, …, *p*_*N*_). This distribution is called the fuzzy *p*-value.

### 2.4. Analysis of Crohn's disease dataset

We applied the tree-based association approach to a publicly-available dataset composed of 258 trios consisting of a father, mother and a child affected with Crohn's disease, originally analysed by Rioux et al. (2001). The genetic data consists of genotypes at 103 SNP markers across 500 kb of the 5q31 region of chromosome 5. The dataset is available either in the R gap package (Zhao, 2013) or at the author's website: http://www.broadinstitute.org/archive/humgen/IBD5/haplodata.html.

Beagle (Browning and Browning, 2009) was first used to impute haplotype phase and missing marker genotypes. We chose Beagle for imputation and phasing because it could handle the size of the dataset and the case-parent trios. The program was run using default settings with the trios option and returned a single estimate of the most likely haplotype that each parent passed to his/her affected child (transmitted) and the haplotypes that were not passed to the child (untransmitted). For this illustration, since our statistic requires two disease groups, we define the transmitted haplotypes as the cases and the untransmitted haplotypes as the controls.

We sampled ancestral trees at 100 focal points spaced evenly throughout the 500 kb region. For each focal point, a subset of the 103 SNPs was chosen for the analysis: all SNPs within a window size of 100 kb around the focal point were included in the dataset for that focal point. If fewer than 20 SNPs were available in the window then the window-specific dataset was expanded to include the closest 20 SNPs to the focal point, so that each dataset had a minimum of 20 SNPs. If there was less than 100 kb between the focal point and the lower or upper edge of the genotyped region, the window size remained the same but the focal point was not centered in each subset. Due to the sparsity and uneven spacing of SNPs in the region, the majority of window-specific datasets had to be expanded to include 20 SNPs.

Apart from the choice of focal point and dataset settings corresponding to the window, all run options and initial conditions were set to be the same for each focal point. The prior distribution for θ, the mutation rate, was chosen to be uniform on (0.0001,10) and θ was initialized to 0.1. The prior distribution for ρ, the recombination rate, was a gamma distribution with shape parameter 1 and scale parameter 0.1. The initial ρ value was set to 0.0004. The total MCMC chain length was 8 million with a burn-in of 4 million iterations; these values were based on visually assessing convergence and mixing with traceplots of sampled values and tree summary statistics such as the time to the most recent common ancestor and the symmetric distance between trees (Robinson and Foulds, 1981). Since the file sizes of sampled trees can become large, only every 10,000th sample was saved.

Each focal point was run on a separate processor in a cluster computing environment. The median time to complete one million iterations on one focal point was 49 h but the maximum time, over the 100 focal points, for these computations was 64 h. Hence, the total time to complete all eight million iterations on all focal points was on the order of three weeks. For each of the returned trees, we used functions from the R phylogenetic package ape (Paradis et al., 2004) to compute tree-based statistics and to sample ancestral trees from the coalescent prior distribution. We also computed more conventional single-locus (Fisher Exact) and haplotype-based association statistics. Haplotype-based TDT analyses were performed using the R TDTHAP package (Clayton and Jones, 1999) with window sizes of 10 SNPs, 20 SNPs and 100,000 bp (to match our tree-based approach). For estimating the *p*-value within each window, we used 100,000 simulations.

## 3. Results

### 3.1. Estimation of the recombination rate, ρ

Sampletrees provides samples of the recombination and mutation rates, ρ and θ, at each focal point. Although for the anticipated applications of our sampler these parameters may not be of primary interest, we would hope that the sampled values are biologically plausible. Therefore, we compared our estimates of the recombination rates in this region to those available in public databases.

Recombination rate estimates computed by Peter Donnelly, Gil McVean and Simon Myers using the coalescent approach in McVean et al. (2004) are available with the Phase I HapMap data (release 16a) (International HapMap Consortium, 2005). These data were downloaded as part of the bulk data download of chromosome five from http://hapmap.ncbi.nlm.nih.gov/. The HapMap recombination rate was converted from cM/Mb to the rate per pair of base pairs, per unit of coalescent time, by noting that for the per generation rate 1 cM/Mb ≈ 10^−8^/bp and taking an effective population size of 10,000 individuals. Although both sets of data cover the same region, the SNP positions provided with the Crohn's dataset were relative to the SNP discovery region and not the genomic positions. Therefore, the two sets of results could not immediately be compared without first finding a mapping between the Crohn's dataset and HapMap positions. The rs numbers for the SNPs were not provided with the Crohn's dataset. To determine the SNP positions in HapMap, we conducted a literature search and found rs numbers for two of the SNPs. We then used the UCSC Genome Browser (http://genome.ucsc.edu/) to locate the genomic positions of these two SNPs relative to the NCBI Build 34 human reference sequence. Although this reference sequence dates to 2003, the markers from HapMap Phase 1 (release 16a) are relative to this build. However, the distance between the two SNPs was different between the provided positions and the genomic positions from UCSC. The order of the SNPs was also reversed in the two sets of positions. Therefore, we caution that the conversion between the two sets of positions may not be completely accurate.

**Figure 2:**
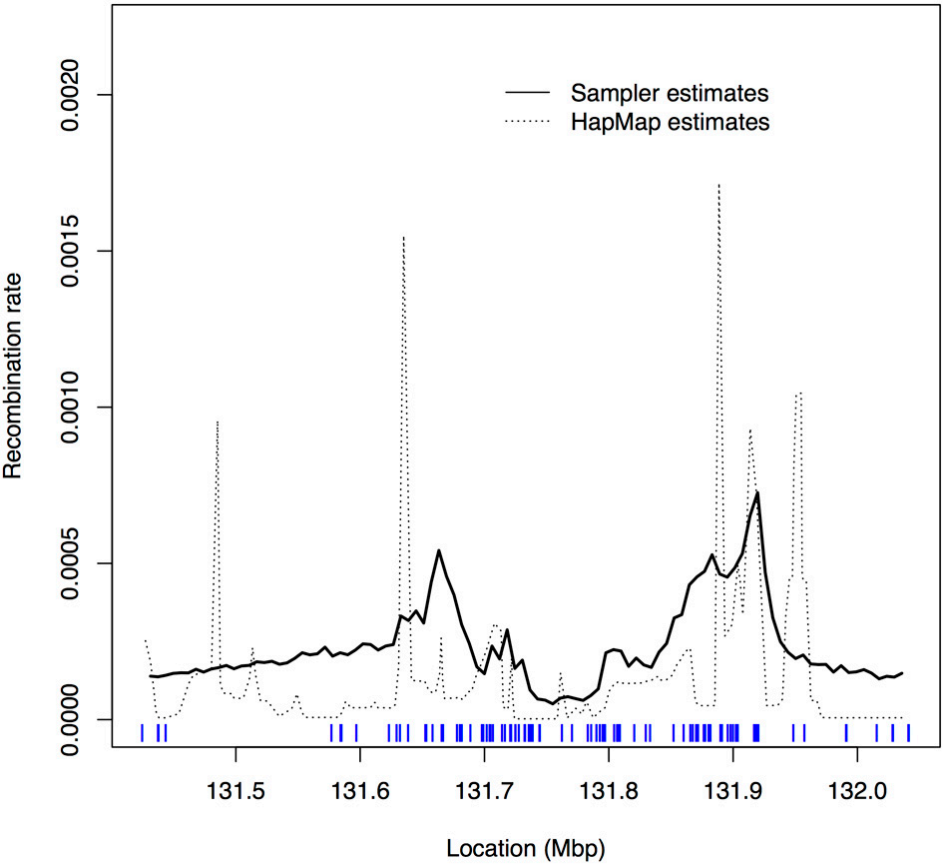
**Plot of recombination rate values, ρ, estimated by sampletrees and by HapMap.** Solid curve: average of the sampled ρ values from sampletrees for each focal point; Dashed curve: rescaled recombination rates estimated from Phase I HapMap data (release 16a) (International HapMap Consortium, 2005). The tickmarks at the bottom show the marker locations.

Figure 2 shows the estimated recombination rates across the region. The dashed curve gives the recombination rates estimated from the HapMap data. The solid curve connects the average of the sampled ρ values from sampletrees at each focal point. The sampletrees estimate for each focal point is based on window sizes of varying numbers of markers and of variably-spaced markers (spacing ranges from 38 to 133,517 kb); therefore, the solid curve should be viewed as a smoothed version of the HapMap estimates (the dashed curve) since our estimates are based on fewer, less equally spaced SNPs than the HapMap estimates. It is therefore not surprising that the peaks are lower and the distribution smoother overall. The shifts in peak locations are possibly due to the difficulties in aligning the HapMap positions with the Crohn's dataset positions. Nevertheless, taking these factors (i.e., smoothing and shifts) into consideration, it is satisfying to verify that the variation in recombination rate estimated by our algorithm is consistent with the variation estimated by others using different data and different algorithms. Although we see two concordant peaks, we do not pick up the peak near 131.5. However, in this dataset there were no markers genotyped in this region, and so there may not be enough genotype information to detect the increase in recombination rate indicated by the HapMap estimates.

### 3.2. Association analysis

**Figure 3:**
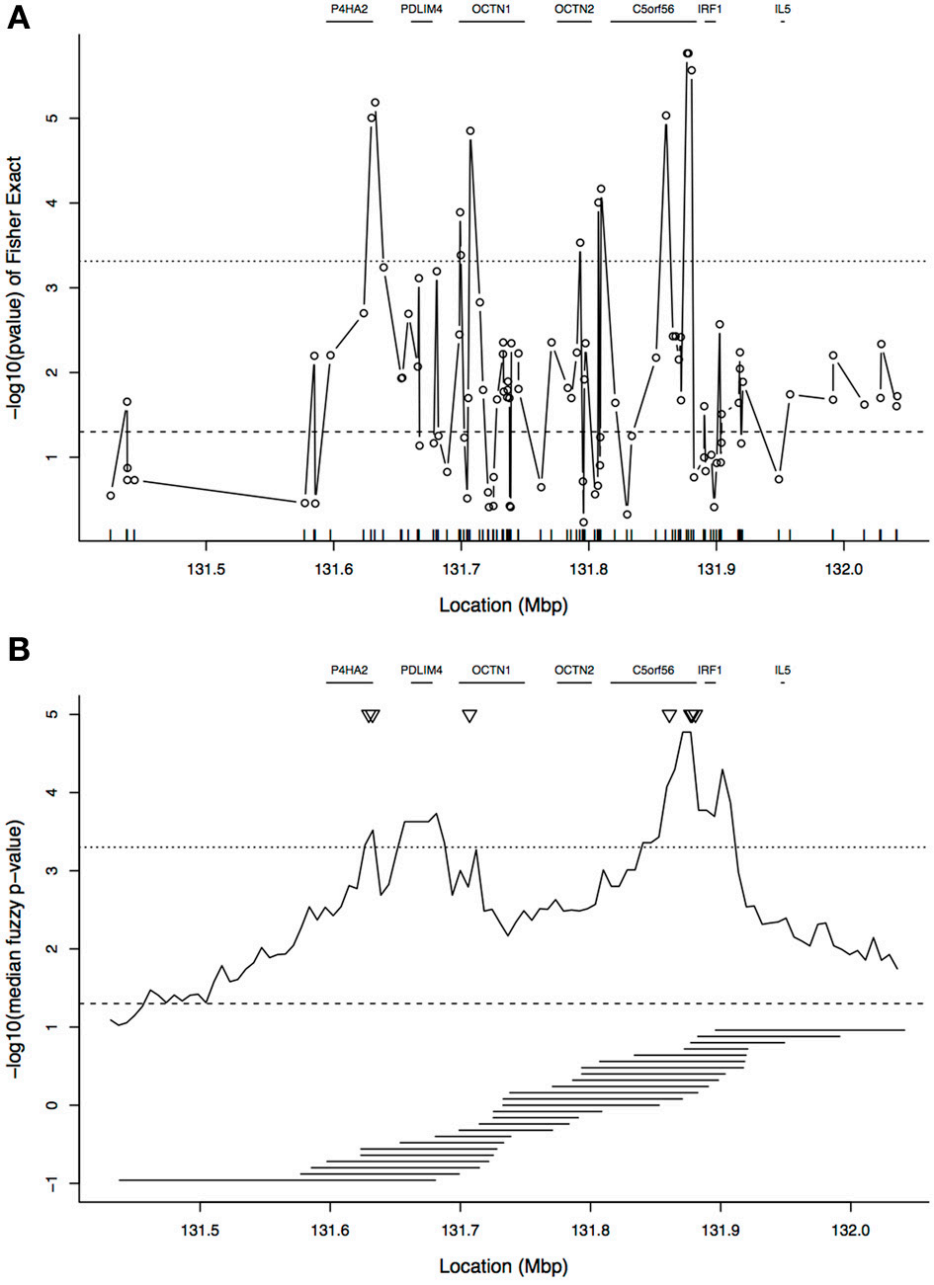
**Plot of association results in the 5q31 region. (A)** Single-SNP analysis: plot shows −log_10_(p-value) from Fisher's exact test of association between allelic state and case/control status. The tickmarks at the base of the plot show the locations of the SNPs. **(B)** Tree-based analysis: −log_10_ of the median of the fuzzy *p*-value by focal point. In **(B)**, the tiled horizontal line segments under the association curve show the window spans for every second focal point. In both panels, gene locations are indicated at the top of each panel. The horizontal dotted line near *y* = 3.3 indicates a *p*-value of 0.05 after Bonferroni correction, and the horizontal dashed line near *y* = 1.3 is the uncorrected *p*-value threshold of 0.05. The Bonferroni correction for **(A)** is based on 103 SNPs and for **(B)** it is based on 100 focal points. The triangles in **(B)** correspond to the peaks of **(A)**.

Figure 3A shows the single-locus association results for these data. At each locus, Fisher's exact test was used to determine whether there was an association between allelic state and case/control status, where “cases” were the transmitted chromosomes and “controls” the untransmitted chromosomes. This figure also shows the locations of genes in the region. As expected from the published results on this region, although there are a few peaks, the signal in this region is not distinct and spans a large region. Many SNPs pass the *p* = 0.05 threshold of significance even if a Bonferroni correction is applied to account for the 103 SNPs tested.

With respect to the ancestral tree of a disease-mutation, we expect cases to preferentially coalesce with each other rather than with the controls, indicating that they are more closely related at that focal point. The increased relatedness of the cases will be reflected by a clustering of case haplotypes in the ancestral tree. At each focal point, for the *j*th sampled tree, we computed *T_j_*, the maximum absolute correlation between disease status and cluster membership, as described in Section 2.2, and the corresponding latent *p*-value, *p_j_*, as described in Section 2.3, with *M* = 35,000 samples from the coalescent prior distribution.

The −log_10_ of the median of the latent *p*-value distribution is given for each focal point in Figure 3B. The signal from the median of the latent *p*-values can be compared to the single-locus results in Figure 3A. In Figure 3B, the peak correlation between disease status and cluster membership, occurring near 131.9 Mbp, is close to a peak of the single-locus results (as indicated by the triangles). A second area of high signal from the cluster-based results is between 131.6 and 131.7 Mbp; however, the peak in this region corresponds to a *p*-value that is approximately 10-fold higher than the peak near 131.9 Mbp. In contrast, in the single-locus results, there are two additional peaks near 131.6 and 131.7 Mbp having height only slightly below the overall peak near 131.9 Mbp. These additional peaks of the single-locus results flank the lower, second peak of the cluster-based statistic. It is evident that the cluster-based statistic yields a smoother association curve than the single-locus results, with more distinct peaks. However, the tree-based results are more than a smoothed version of the single-locus results because they de-prioritize the two single-locus peaks near 131.6 and 131.7 Mbp. We return to this point in the Discussion.

**Figure 4:**
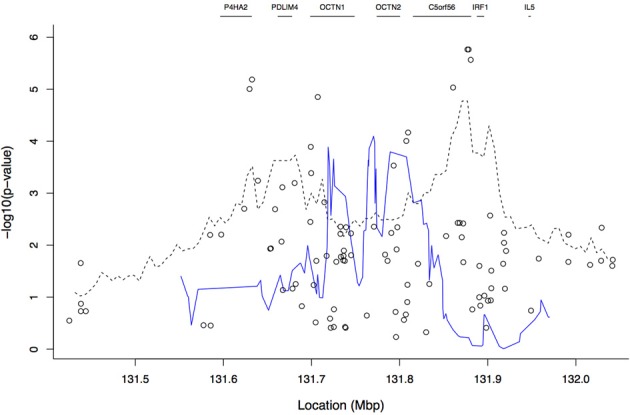
**Plot of −log_10_ of the *p*-values from the TDTHAP analysis using a window size of 20 SNPs (blue solid line).** The open circles and the dashed line give the single-SNP and tree-based results, respectively, that were also shown in Figure 3. Gene boundaries are marked by horizontal line segments at the top of the plot.

Figure 4 shows the results from the TDTHAP analysis with a window size of 20 SNPs, along with those from the tree-based and single-locus analyses. The TDTHAP results differ from both the single-SNP and tree-based results, though the peak location of the TDTHAP results in the *OCTN1*/*OCTN2* region is more compatible with the single-SNP analyses. TDTHAP appears to be sensitive to window size; the results with 10 SNPs were more erratic, while with the 100,000 bp window size the peaks had all been smoothed out (not shown).

**Figure 5:**
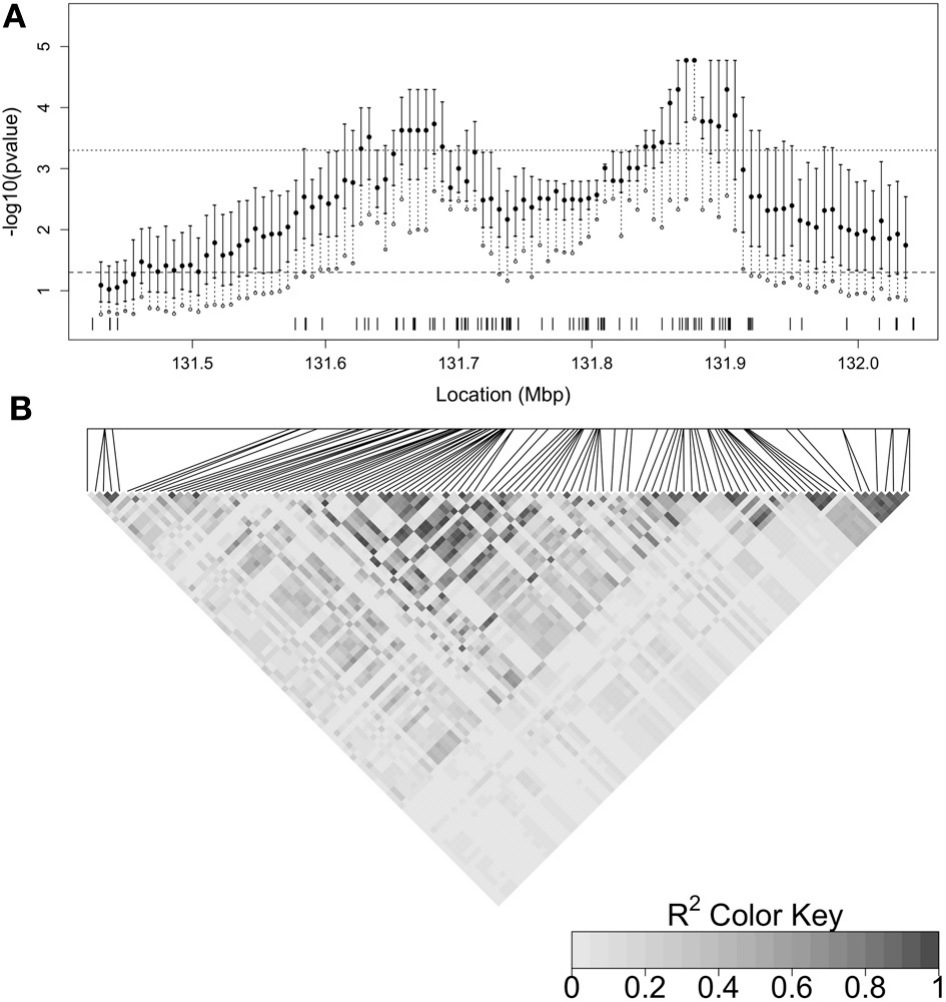
**(A)** Plot summarizing the distribution of the latent *p*-values by focal point. The inter-quartile range (IQR) of the latent *p*-values at each focal point is indicated by the solid vertical line. The filled in circle is the median and the open circle is the 90th percentile of the distribution. The dashed vertical line therefore indicates the range from the 75th to 90th percentile. The dashed horizontal line indicates a *p*-value cutoff of 0.05 and the dotted horizontal line shows a *p*-value cutoff of 0.0005 (0.05, Bonferroni-corrected for 100 focal points). SNP locations are marked by tickmarks at the base of the plot. **(B)** Heatmap of linkage disequilibrium (*R*^2^) between SNPs estimated from control haplotypes and displayed by LDheatmap (Shin et al., 2006). The relative positions of the SNPs are given by the horizontal line above the heatmap and the positions are aligned with **(A)**.

Figure 5 summarizes the distribution of the latent *p*-values for each focal point and can be used to evaluate the uncertainty associated with the latent genealogy. The degree of uncertainty is not the same at each focal point, as indicated by the width of the inter-quartile range (IQR). In general, the width is larger when there are fewer nearby SNPs, and hence less information in the marker data about the latent tree (Thompson and Geyer, 2007). The effect of marker density can be seen, for example, when comparing the widths near 131.9 to 132.0 Mbp. The width of the interval is smaller at the peak of the cluster-based results but this may be due to an inadequate number of samples from the coalescent prior distribution for estimating the low *p*-values in this region. In particular, since there were *M* = 35,000 samples from the prior distribution, any latent *p*-values of zero have been set to $\frac{1}{35000}$ to enable plotting on the log scale.

**Figure 6:**
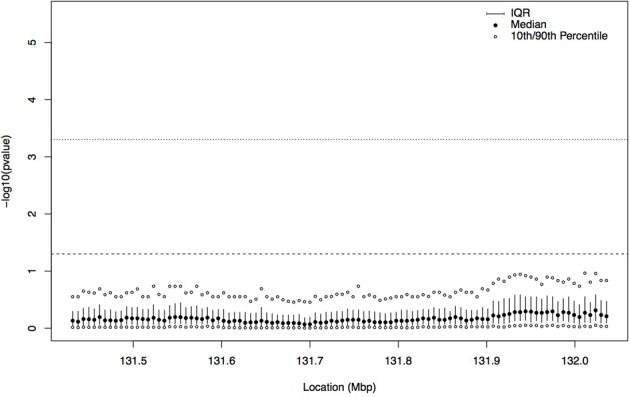
**Plot summarizing the distribution of the latent *p*-values by focal point for the permuted case-control labels on haplotypes.** The interquartile range (IQR) at each focal point is indicated by the solid vertical line, the filled in circle is the median, and the open circles represent the 10th and 90th percentiles of the distribution of −log_10_ of the latent *p*-values. The dashed horizontal line indicates a *p*-value cutoff of 0.05 and the dotted horizontal line shows a *p*-value cutoff of 0.0005 (Bonferroni corrected for 100 focal points). For all focal points, the 90th percentile of the distribution of −log_10_ of the latent *p*-values is below the 0.05 cutoff.

In order to gain insight about whether the association signal from the tree-based analysis of this region could be a false positive result, we repeated the analysis with a dataset consisting of a permutation of the case-control labels versus the haplotype data. Since the tree sampling step does not use phenotype information, we simply computed the correlation statistic between the previously sampled trees and the permuted phenotypes. The distribution of latent *p*-values from the permutation, at each focal point (as described in Section 2.3), can be seen in Figure 6. For the permuted phenotype, there is no evidence of association at any location in this region; across all focal points, the 90th percentile of −log_10_ of the fuzzy *p*-value is always less than the uncorrected 0.05 cutoff (the dashed horizontal line), and hence, as expected, there is no evidence that case haplotypes are clustered together in ancestral trees in the region.

## 4. Discussion

In this work, we have described an ancestry-based approach to association mapping that accounts for the uncertainty of the ancestral tree at a given genomic location. With this approach, multi-marker SNP data is used to sample ancestral trees from their posterior distribution under the neutral coalescent. Each sampled tree is used to define clusterings of the tips and the association is tested using the maximum correlation between cluster membership and disease status. We showed how to compute the fuzzy *p*-value with the neutral coalescent as the prior distribution in order to assess the evidence for association and the uncertainty due to the latent ancestral tree. We emphasize that due to the computational needs of this approach, the ancestry-based approach would be proposed for fine-mapping and would therefore be applied to a gene-region that has already been identified through, for example, a linkage study or a GWAS. This approach requires that genotype data be available for multiple linked markers in the identified region, as it is the pattern of allelic association between the markers that provides information about the underlying ancestral tree.

We illustrated the approach using the publicly-available 5q31 dataset of case-parent trios with Crohn's disease. We first imputed haplotype phase using the family information to estimate the transmitted and untransmitted haplotypes from parents to affected offspring. We then sampled ancestral trees and recombination rates at 100 focal points across the 500 kb region to compare transmitted and untransmitted haplotypes. Mixing and convergence were assessed with traceplots of sampled values and tree summaries (results not shown).

We compared our estimates of the recombination rates in this region to those estimated by HapMap and found concordant estimates. However, our recombination rate estimates were typically lower than those of HapMap and the overall curve appeared smoother, which can be explained by the variable window size and SNP density that was available in this dataset. The recombination rate estimated by sampletrees is the rate per adjacent pair of base pairs, but this rate is assumed to be constant across the window. The estimate is therefore the average recombination rate (per adjacent pair of base pairs) across the window. Unfortunately, the SNPs available in the Crohn's 5q31 dataset were very unevenly spaced, particularly at the edges of the region. The uneven spacing led to some windows spanning large physical distances and having variable recombination rates across the window. For these windows, the estimated recombination rate from sampletrees is therefore averaging this variable rate over these large distances, leading to a smoother curve than the HapMap results.

We then computed the fuzzy *p*-value of the ancestry-based association statistic at each focal point. Examination of the median of the fuzzy *p*-value across focal points showed that the maximum peak locations were close to the single-locus association results previously published; however, the cluster-based results appear smoother, and the peak is more distinct than in the single-SNP analysis. In the tree-based analysis, the *p*-value in *C5orf56* near *IRF1* is approximately 10-fold smaller than any other areas of peak signal away from *IRF1* (such as *PDLIM4*). In contrast, for the single-SNP analyses the *C5orf56* signal near *IRF1* is only slightly enhanced relative to the signals near *P4HA2* and *OCTN1*. The tree-based analysis de-prioritizes the single-locus analysis signal near *P4HA2* and *OCTN1*. Therefore, the tree-based analysis is not just smoothing the single-locus results; if it were, we would expect the peak near *IRF1* to be diminished like the *PDLIM4* signal. The tree-based analysis also prioritizes different regions than the haplotype-based analysis. The peak region for TDTHAP is near *OCTN1* and *OCTN2*, between the two single-locus peaks, which may be due to the best haplotype window picking up association of both single-locus peaks simultaneously. To summarize, the tree-based approach indicates that the transmitted haplotypes are more genetically related than the untransmitted haplotypes and that there may be one or multiple disease-predisposing loci in the region. Although not examined here, evaluation of how the window size and local LD patterns affect the behavior of the association statistic is an interesting question for future research.

Because the Crohn dataset is publicly available, many groups have used it to evaluate newly developed methodologies. Conti and Witte (2003) developed a two-stage analytic approach that modeled the odds ratio from a TDT analysis of each SNP with a random effects model having means that depended on haplotype block membership. They compared their approach to the single-SNP analysis and found similar results. Zheng and McPeek (2007) developed a multi-point mapping method that also made use of haplotype blocks; when applied to the Crohn dataset, the same 9 significant SNPs from the original analysis in Rioux et al. (2001) remained significant and two more SNPs reached region-wide significance. Browning (2006) used this dataset to illustrate the Variable Length Markov Chain (VLMC) technique. Although the major features of the single-SNP results, including the significant extended haplotype, were seen with the VLMC analysis, it did not provide additional insights about the location of disease pre-disposing loci. Therefore, although this dataset has been analyzed with several approaches that are richer or more sophisticated methodologies than single-SNP analyses, these analyses have not necessarily provided additional insights beyond those from the original analysis by Rioux et al. (2001).

Unfortunately, determining which variant(s) explain the association signal has proven to be difficult due to the strong LD observed in this region. The risk haplotype, IBD5, spans a 200 kb region containing multiple genes, as shown in Figure 3. Peltekova et al. (2004) found that two SNPs in the *OCTN1* and *OCTN2* genes were associated with inflammatory bowel disease (IBD), with Crohn's disease a major subtype of IBD, independent of the risk haplotype. However, subsequent studies did not replicate this finding. Nevertheless, these two genes, and specifically the L503F variant in *OCTN1*, are believed to be good candidates due to their role in maintaining barrier function in the intestine (Barrett and Chandra, 2011). In our results, we do not see a high signal from either of these two genes.

The peak signal in our results is near the *IRF1*/C5orf56 region. Recently, two papers examining selection in the IBD5 region have also pointed to this subregion as harboring IBD variants rather than the *OCTN1*/*OCTN2* genes. Cagliani et al. (2013) cross-categorized SNPs identified by a genome-wide association study of IBD with SNPs showing patterns of selection to pathogens. Of 43 IBD-associated SNPs, eight showed a strong link with selection due to protozoa, including rs2188962 in the C5orf56 region. Huff et al. (2012) suggested that the immune-related *IRF1* gene is a better candidate gene for association with IBD than the other genes in the region. They argued that association of IBD with variants in *OCTN1* is actually explained by selection of the *OCTN1* L503F variant. This variant increases transport of ergothioneine, causing the true IBD-predisposing variant in a nearby gene to also reach higher frequencies (genetic hitchhiking); the *IRF1* gene is 0.057 cM away from the L503F variant. They also argued that positive selection on variants in this region explains the unusually complex pattern of LD that has been documented. To further support *IRF1* as the candidate gene for IBD association, they showed that haplotypes having evidence of recombination between L503F and *IRF1* are not associated with IBD whereas haplotypes that have no evidence of recombination are associated with IBD. Our results, which show the highest association near *IRF1*, are consistent with both of these works.

In the analysis presented, we used a single imputation of haplotypes based on the trio data. Bias of the haplotypic odds ratio, inflated type I error rates and low power have all been observed in haplotype-based association studies using single imputation of haplotypes (Lin and Huang, 2007; Mensah et al., 2007). However, our haplotype estimates are based on the family trios, and therefore the imputed haplotypes are likely closer to the true values than when imputation is done with samples of unrelated individuals. Although we have implemented a version of sampletrees that handles missing haplotype phase (Burkett et al., 2013b), it does not currently utilize the phase information available from the family data. In the future, we would like to extend sampletrees to handle partially-known haplotypes, as would be available from trio data, for example.

A tree-based approach is flexible in that other test statistics can be defined to capture different underlying disease models. We have presented a tree-based association statistic that clusters the data into two groups, and would be expected to be optimal for a single-disease predisposing mutation that is relatively common. All descendants of the internal branch on which the mutation took place would carry the mutation and be part of the same group for the bipartition formed by that branch. However, we are currently investigating the potential utility of a tree-based approach for the discovery of a set of rare variants associated with disease. In a tree-based approach, rather than evaluating association of alleles at rare variants with disease status, the test statistic would capture increased relatedness of groups of haplotypes derived from case individuals, with each group corresponding to a different rare variant.

### Conflict of interest statement

The authors declare that the research was conducted in the absence of any commercial or financial relationships that could be construed as a potential conflict of interest.
